# Retinitis Pigmentosa Associated with Glucose-6-Phosphate Dehydrogenase Deficiency

**DOI:** 10.7759/cureus.1506

**Published:** 2017-07-23

**Authors:** Bryan Thiel, Aman Sharma, Saad Shaikh

**Affiliations:** 1 University of Central Florida College of Medicine; 2 Ophthalmology, Orlando Veterans Affairs Medical Center; 3 Ophthalmology, University of Central Florida College of Medicine

**Keywords:** retinitis pigmentosa, glucose-6-phosphate dehydrogenase deficiency, oxidative, metabolism

## Abstract

We report a case of new onset retinitis pigmentosa (RP) associated with a glucose-6-phosphate dehydrogenase (G6PD) deficiency in a 63-year-old African-American male who presented with worsening night vision over a period of five years. The pathogenesis of G6PD-mediated oxidative biological damage is reviewed and a mechanism for the onset of retinal disease proposed.

## Introduction

Retinitis pigmentosa (RP) is a group of dystrophies inherited in a dominant, recessive, or X-linked manner and characterized by the degeneration of retinal photoreceptor and pigment epithelial cells. The outer rod cells, important for achromatic night vision, are primarily affected, leading to night blindness followed by diminished peripheral vision. As RP progresses, the inner cone cells begin to degenerate, leading to visual field deficits and central blindness [1].

The glucose-6-phosphate dehydrogenase (G6PD) deficiency is an X-linked recessive disorder characterized by a defect in the G6PD enzyme leading to the oxidative injury of red blood cells (RBCs). While most individuals are asymptomatic, affected males may present with hemolytic anemia, hemoglobinuria, and jaundice. G6PD is an enzyme that is crucial for the formation of nicotinamide adenine dinucleotide phosphate (NADPH), which is an important substrate in the pentose phosphate pathway. NADPH functions to regenerate glutathione. Glutathione, an antioxidant, protects cells in the body from oxidative damage but becomes oxidized in the process [2]. A deficiency of G6PD leads to a deficiency of NADPH and the inability to regenerate glutathione, leaving RBCs susceptible to oxidative injury. This is the predominant mechanism that leads to erythrocyte lysis, intravascular hemolysis, and the clinical manifestations described earlier. This case highlights several proposed mechanisms of RP and how G6PD deficiency may accelerate the dystrophic conditions of the photoreceptor cells of the retina.

## Case presentation

**Figure 1:**
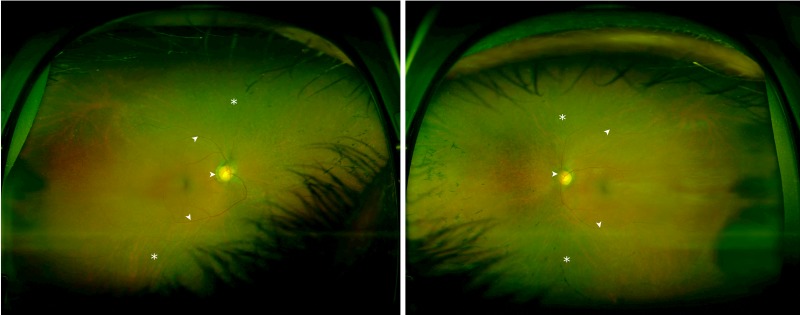
Color Fundus Photographs Pigment clumps are dispersed throughout the peripheral retina in a perivascular pattern (asterisks). Arteriolar narrowing and optic disc pallor are noted bilaterally (arrows).

A 63-year-old African-American man with a G6PD deficiency, type 2 diabetes mellitus, hyperlipidemia, essential hypertension, and adenocarcinoma of the prostate status post resection in March 2016 presented with complaints of worsening night vision over the past five years. There was no family history of RP, nyctalopia, peripheral or central vision loss, or blindness. His past medication use included anti-malarial treatment for several months during active military duty. Visual acuity was 20/30+2 in the right eye and 20/30-2 in the left eye. Intraocular pressures were within normal limits bilaterally. A dilated funduscopic examination demonstrated bilateral retinal pigmentary changes along with chorioretinal degeneration, vascular attenuation, and optic nerve pallor (Figure 1).

**Figure 2:**
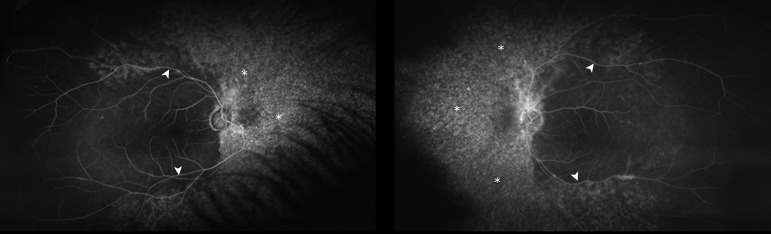
Fluorescein Angiography Widespread retinal pigment epithelial disruption is noted with atrophy and staining (asterisks). Retinal vascular attenuation is also present bilaterally (arrows).

An ocular coherence tomography showed normal foveal contour and thickness bilaterally. An intravenous fluorescein angiography demonstrated background peripheral hypofluorescence/hyperfluorescence correlating with chorioretinal atrophy (Figure 2).

**Figure 3:**
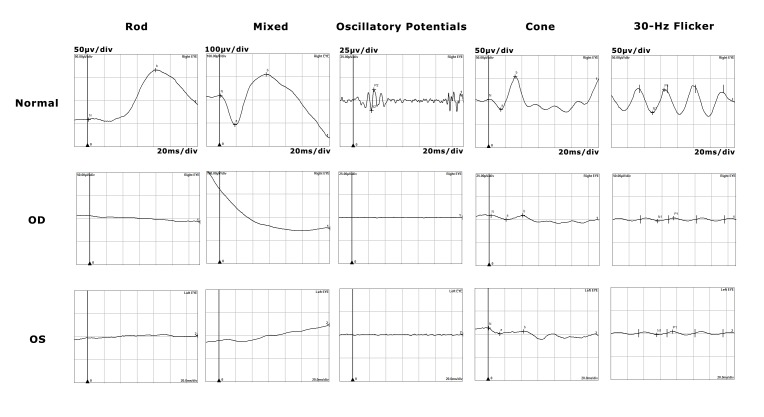
Full-Field Electroretinogram Extinguished scotopic responses and oscillatory potentials along with markedly diminished photopic and flicker responses are noted bilaterally (middle and bottom row of images). A reference normal electroretinogram is displayed for clinical comparison (top row).

A standardized Humphrey visual field examination confirmed significant field constriction. A magnetic resonance imaging (MRI) scan of the brain and orbit demonstrated moderate diffuse cerebral atrophy and scattered foci in periventricular white matter changes but was otherwise unremarkable. The multifocal electroretinogram demonstrated decreased amplitude, right eye more so than left with normal latency. A full-field electroretinogram demonstrated nearly extinguished scotopic responses, decreased oscillatory potential (OCP) amplitudes, and diminished photopic and flicker responses in both eyes (Figure 3).

Laboratory studies demonstrated deficient G6PD levels. The anti-recoverin antibody, rapid plasma reagin (RPR), and quantiferon gold tests were negative. The thiamine (B1), pyridoxine (B6), folate (B9), and cyanocobalamin (B12) levels were all within normal levels. The patient was diagnosed with primary RP and started on daily lutein 12 mg tablets and vitamin A palmitate 15,000 IU to reduce the rate of degeneration of photoreceptor cells.

## Discussion

Several case reports have established a relationship between cataracts and G6PD deficiency, but only one study has previously linked this disease with RP [3]. In the latter, Zeng et al. demonstrated that the incidence of G6PD deficiency was higher in patients with RP than in the general population in China, concluding that there may be a biochemical relationship between these two diseases [4]. One proposed mechanism for cataract formation in a G6PD deficiency is similar to that of erythrocyte lysis. The avascular lenses contain crystalline proteins that are susceptible to oxidative injury and, as a result, there is a requirement of high levels of (chemically) reduced glutathione to maintain protein integrity [5]. As a direct result of depleted NADPH, reduced glutathione is not formed, resulting in insoluble crystalline protein aggregates and the formation of cataractous lenses. This principle of oxidative injury in the context of a G6PD deficiency can be applied to the pathophysiology of RP.

We surmise that in patients with a G6PD deficiency, the inability to eliminate oxidants as a result of lower levels of reduced glutathione contributes to the oxidative injury of the retinal photoreceptor cells [6-7]. It has been demonstrated that in patients with RP, there is a significant decrease in the ratio of reduced to oxidized glutathione in the aqueous humor [8]. A depletion of NADPH, a co-molecule necessary for the conversion of all-trans retinal to all-trans retinol (or Vitamin A) in the visual cycle, may also have contributed to the pathogenesis of RP in this patient, as has been noted in the rhodopsin (RHO) and retinal pigment epithelium-specific 65 kDa protein (RPE65) forms of RP [9].

## Conclusions

This case report elucidates the possibility of oxidative stress-induced RP because of the decreased free-radical scavenging resulting from a G6PD deficiency. Ophthalmologists should be aware of the association of RP and G6PD deficiencies in patients presenting with nyctalopia or diminished visual field perception.
